# Examining the Effects of Motorcyclist Risk Behavior and Protective Behavior on Motorcycle Crash Involvement

**DOI:** 10.3390/ijerph23070897

**Published:** 2026-07-12

**Authors:** Dissakoon Chonsalasin, Thanapong Champahom, Sajjakaj Jomnonkwao, Vatanavongs Ratanavaraha

**Affiliations:** 1 Department of Transportation, Faculty of Railway Systems and Transportation, Rajamangala University of Technology Isan, Nakhon Ratchasima 30000, Thailand; dissakoon.ch@rmuti.ac.th; 2 Department of Management, Faculty of Business Administration, Rajamangala University of Technology Isan, Nakhon Ratchasima 30000, Thailand; 3 School of Transportation Engineering, Institute of Engineering, Suranaree University of Technology, Nakhon Ratchasima 30000, Thailand; sajjakaj@g.sut.ac.th (S.J.); vatanavongs@g.sut.ac.th (V.R.)

**Keywords:** motorcycle safety, rider behavior, MRBQ, protective behavior, structural equation modeling, crash involvement, multigroup analysis

## Abstract

**Highlights:**

**Public health relevance—How does this work relate to a public health issue?**

- Motorcyclists are disproportionately represented in road-traffic deaths and serious injuries worldwide, particularly in low- and middle-income countries where powered two-wheelers dominate and riders cluster among younger, lower-income groups.
- Because crashes fall heavily on working-age earners, motorcyclist safety is both an injury-prevention and a social-equity issue, carrying direct medical costs and indirect costs to productivity and household welfare.

**Public health significance—Why is this work of significance to public health?**

- In a large sample of 2910 active riders, an integrated mixed-theory model (HIP, GEMS, TPB, PMT) shows that risk behavior is associated with higher crash involvement (β = 0.309), while protective behavior is associated with lower crash involvement (β = −0.090), capturing both crash occurrence and injury mitigation.
- The harmful effect of risky riding is roughly three times the protective effect of safety behavior. The association of risky riding with crash involvement is roughly three times as strong as that of protective behavior. These associations are moderated by generation; risky riding is more strongly associated with crash involvement among younger (Gen Z) riders, whereas protective behavior is more strongly associated with lower crash involvement among older (Gen Y) riders.

**Public health implications—What are the key implications or messages for practitioners, policy makers and/or researchers in public health?**

- Programs should be two-pronged: suppressing risky riding through hazard-perception/skills training plus enforcement-backed attitude change while promoting protective-equipment use through PMT-based threat-and-efficacy messaging.
- Interventions should be generationally tailored—risk-suppression for younger riders, protection-reinforcement for older riders—so limited resources target the greatest marginal impact.

**Abstract:**

(1) Background: Motorcyclists remain disproportionately represented in road-traffic fatalities and serious injuries worldwide, yet the behavioral factors associated with their crash involvement are still incompletely understood. (2) Methods: This study integrates several established behavioral theories—Human Information Processing (HIP), Reason’s Generic Error-Modelling System (GEMS), the Theory of Planned Behavior (TPB), and Protection Motivation Theory (PMT)—into a single mixed-theory framework in order to examine simultaneously how risk behavior and protective behavior are associated with self-reported motorcycle crash involvement. A cross-sectional survey was administered to 2910 active motorcyclists using a Modified Motorcycle Rider Behavior Questionnaire (MRBQ) to capture four dimensions of risk behavior. (3) Results: A second-order confirmatory factor analysis (CFA) confirmed that the four risk dimensions load onto a single higher-order motorcyclist risk behavior construct, and the full measurement model demonstrated good reliability, convergent validity, and discriminant validity. Structural equation modeling (SEM) showed excellent fit. Motorcyclist risk behavior was positively and significantly associated with crash involvement, whereas protective behavior was negatively associated with it; because protective equipment mainly reduces injury severity rather than preventing crashes, this inverse relationship is interpreted as an indirect association rather than a direct reduction in crash occurrence, and both hypotheses were supported. (4) Conclusions: The findings support the value of integrating error-based and motivation-based theories when modeling motorcyclist safety and highlight the need for generationally tailored interventions that simultaneously reduce risky riding and promote consistent protective behavior.

## 1. Introduction

Road-traffic injury is one of the leading causes of preventable death globally, and motorcyclists carry a share of that burden that is far out of proportion to their numbers. Powered two-wheelers offer affordability, fuel economy, and maneuverability in congested traffic, which have made them a dominant mode of transport across much of Asia, Africa, and Latin America. The same characteristics that make motorcycles attractive—light weight, limited physical protection, and exposure of the rider’s body—also make their users among the most vulnerable participants in the road system [1,2,3]. When a motorcycle is involved in a collision, the absence of a protective shell means that even comparatively low-energy impacts can result in severe or fatal injury. In many low- and middle-income countries, motorcyclists account for a substantial fraction of all road deaths, and in some settings the majority of traffic casualties involve a powered two-wheeler [4,5].

Beyond the human cost, motorcycle crashes impose a heavy and recurring economic burden. The direct costs of emergency response, hospitalization, rehabilitation, and long-term disability care, together with the indirect costs of lost productivity, premature mortality, and reduced household earnings, fall disproportionately on working-age adults who are often the primary earners in their families [6]. Because motorcyclists in many economies are concentrated among younger and lower-income groups, the welfare consequences of each crash ripple outward to dependents and communities, deepening cycles of disadvantage. These distributional features make motorcyclist safety not only a clinical and engineering challenge but also a question of social equity, and they raise the stakes of identifying the behaviors and rider groups most amenable to intervention.

The persistence of this toll despite decades of engineering improvements, legislative reform, and enforcement campaigns points to the central role of human behavior [7]. Vehicle design and road infrastructure set the boundary conditions of safety, but the moment-to-moment decisions and habits of the rider determine how often those boundaries are tested. Behavioral epidemiology of road crashes has consistently identified human factors—errors of perception and control, deliberate violations of traffic rules, and the non-use of protective equipment—as proximal contributors to crash occurrence and injury severity [8,9]. For motorcyclists in particular, behavior matters twice: first in determining whether a crash occurs at all, and second in determining whether the rider survives it. This dual pathway distinguishes motorcyclist safety research from much of the four-wheel literature and motivates the simultaneous study of behaviors that influence crash involvement and behaviors that influence crash consequences.

A large body of research has applied behavioral instruments to understand why some riders crash more than others. The most widely used of these is the Motorcycle Rider Behavior Questionnaire (MRBQ), an adaptation of the original Driver Behavior Questionnaire designed to capture the specific error and violation patterns of powered-two-wheeler users [10,11]. Studies employing the MRBQ across diverse national contexts have repeatedly shown that self-reported errors and violations are associated with elevated crash risk, and that distinct behavioral dimensions—such as control errors, speeding, and safety-rule violations—can be reliably distinguished [12]. Recent applications in Pakistan and Indonesia, for example, have demonstrated that young riders display a high prevalence of risky behaviors, that lower educational attainment and the absence of a valid license are associated with greater crash involvement, and that “speed” and “errors” are particularly strong correlates of crashes among young riders [13,14]. These findings underscore both the value of the MRBQ and the importance of examining heterogeneity across rider subgroups.

Despite this rich descriptive base, two important gaps remain. First, most studies treat rider behavior as a collection of loosely related factors rather than integrating them within an explicit theoretical architecture that explains why particular behaviors arise and how they combine to produce crashes [15]. Errors of perception, errors of vehicle control, deliberate risky riding, and rule violations are psychologically distinct phenomena: some reflect failures of information processing, others reflect breakdowns in skilled action, and still others reflect motivated choices shaped by attitudes and perceived norms. Treating them as interchangeable indicators obscures their different origins and the different interventions they require. Second, the literature has tended to concentrate on behaviors that cause crashes (risk behavior) while giving comparatively less structured attention to behaviors that protect riders from harm (protective behavior such as helmet and protective-gear use), and rarely models the two within the same framework [16,17,18]. Because risk and protection operate through different psychological pathways—one through error and motivation to offend, the other through threat appraisal and motivation to protect—a complete behavioral account requires both.

**Figure 1:**
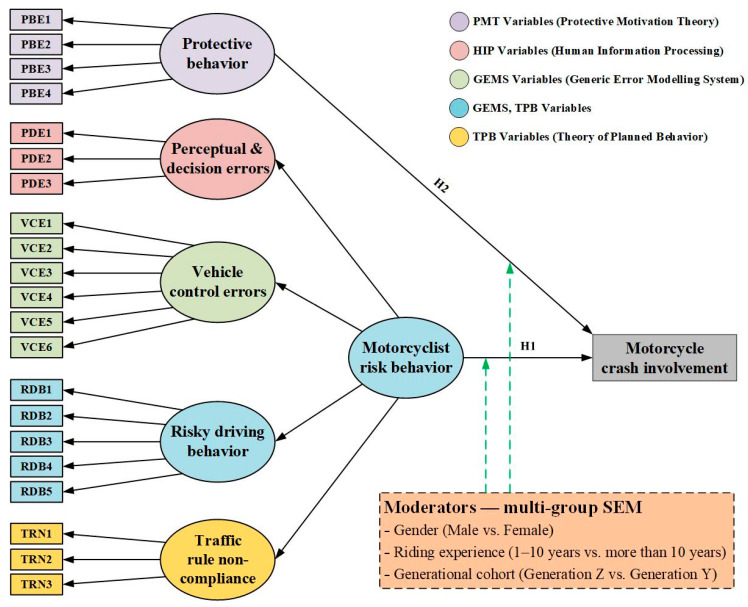
Conceptual Framework.

To address these gaps, the present study adopts a mixed-theory approach. Rather than relying on a single behavioral model, it draws together four complementary theoretical lenses, each suited to a different facet of the behavioral landscape. Human Information Processing (HIP) theory frames perceptual and decision errors as failures at the detection, perception, and decision stages of the riding task [6]. Reason’s Generic Error-Modelling System (GEMS) frames vehicle control errors as breakdowns in skill-, rule-, and knowledge-based performance [19]. The Theory of Planned Behavior (TPB) frames risky riding and traffic-rule non-compliance as motivated, intentional acts shaped by attitudes, subjective norms, and perceived behavioral control [16]. Finally, Protection Motivation Theory (PMT) frames the use of helmets and protective equipment as a coping response driven by threat appraisal and the motivation to protect oneself [20]. Integrating these theories allows the study to model motorcyclist risk behavior as a single higher-order construct built from theoretically grounded sub-dimensions, while modeling protective behavior as a conceptually separate, motivation-based construct (Figure 1).

The empirical setting is a large sample of active motorcyclists for whom both behavioral profiles and self-reported crash histories were collected. Using these data, the study pursues three objectives. The first objective is to validate a measurement model in which motorcyclist risk behavior is represented as a second-order latent construct comprising perceptual and decision errors, vehicle control errors, risky riding behavior, and traffic-rule non-compliance, alongside protective behavior as a distinct construct. The second objective is to estimate the simultaneous effects of risk behavior and protective behavior on motorcycle crash involvement within a structural equation model, testing the expectation that risk behavior increases, and protective behavior decreases, the likelihood of crash involvement. The third objective is to examine whether these structural relationships are stable across key rider subgroups—gender, riding experience, and generational cohort—through multigroup analysis, thereby identifying whether and where interventions need to be tailored.

This study makes three contributions that are deliberately ordered by centrality. The primary contribution is conceptual: the present study is among the first to model motorcyclist risk behavior and protective behavior simultaneously within a single, theoretically integrated framework, thereby capturing both the behaviors that contribute to crash occurrence and the behaviors that influence crash consequences—two pathways that prior MRBQ research has typically examined in isolation [21]. This integration is precisely what the combination of error-based theories (HIP, GEMS) and motivation-based theories (TPB, PMT) within one well-fitting structural model is intended to achieve. The second contribution is a measurement contribution that serves the first: motorcyclist risk behavior is validated as a second-order latent construct comprising perceptual and decision errors, vehicle control errors, risky riding behavior, and traffic-rule non-compliance, providing the parsimonious higher-order representation required to weigh risk against protection within the same model. The third contribution is applied: multigroup analysis establishes the boundary conditions of these relationships across gender, riding experience, and generation, showing that the risk–protection structure is moderated by generational cohort and thereby indicating where interventions need to be tailored. Framed in this way, the second-order specification and the generational analysis are not independent claims but, respectively, the measurement basis for and the boundary test of the study’s central novelty—the simultaneous, theory-based modeling of risk and protective behavior.

## 2. Theoretical Background and Hypothesis Development

### 2.1. The Behavioral Approach to Motorcyclist Crash Risk

The behavioral tradition in road-safety research holds that, once vehicle and environmental conditions are accounted for, the recurring driver of crash variation is what road users themselves do. Within this tradition, behaviors are commonly divided into errors—unintended deviations of action from intention—and violations—deliberate departures from safe or legal practice [11]. This distinction, originally formalized for car drivers, has proven equally informative for motorcyclists, whose behavioral repertoire includes both unintentional lapses in perception and control and intentional acts such as speeding, red-light running, and riding without protective equipment. The MRBQ operationalizes this repertoire, and the present study builds on it by organizing the resulting behavioral dimensions according to the theoretical mechanisms that generate them.

A key premise of this study is that the various risky behaviors captured by the MRBQ are not theoretically homogeneous. Some arise from limitations in how riders take in and process information; others arise from breakdowns in the execution of skilled riding actions; and still others arise from motivated decisions to break rules. Lumping them together can be statistically convenient but theoretically misleading, because each class of behavior has a different psychological origin and therefore responds to different countermeasures. The mixed-theory framework adopted here makes these origins explicit by assigning each behavioral dimension to the theory best equipped to explain it, while still allowing the dimensions to combine into an overarching propensity toward unsafe riding.

### 2.2. Prior Empirical Evidence from the MRBQ Tradition

The MRBQ has now been applied in a wide range of countries, and a consistent set of empirical regularities has emerged that motivates the present specification. Studies repeatedly recover a multi-factor structure separating control errors, speed-related violations, traffic errors, and the use—or non-use—of safety equipment, indicating that motorcyclist behavior is not unidimensional [22,23]. Across these settings, self-reported errors and violations are positively associated with crash and near-crash involvement, supporting the predictive value of the instrument [24,25]. Recent work in South and Southeast Asia is particularly relevant to the present context. Studies of young motorcyclists in Pakistan using a Modified MRBQ have identified safety violations, speeding, traffic errors, stunts, and control errors as distinct factors, with safety violations emerging as the most significant correlate of risk and with lower educational attainment, the absence of a valid license, larger engine capacity, and longer riding exposure all associated with greater crash involvement [13,26]. Comparable research in Indonesia, where motorcycle dependence among young people is high, has found that riders aged 15–19 have the highest crash involvement, that many ride unlicensed or before the legal age, and that “speed” and “errors” are especially strong predictors of crashes among the young [14,27]. Naturalistic and simulator studies add an observational complement to the questionnaire findings: analyses of safety-critical events confirm that human error is the leading cause of motorcycle incidents [28], and experimental comparisons of advanced, experienced, and novice riders show that training is associated with lower speed violations, fewer traffic errors, better lane positioning, and faster hazard response [29]. Evidence from Vietnam and India similarly links risky ridership and traffic errors to elevated crash risk [20,30,31].

Regional evidence from Southeast Asia further reinforces both the psychological heterogeneity of rider behavior and its link to safety outcomes. In Indonesia, a study of online motorcycle-taxi drivers in Semarang used partial least squares structural equation modeling to relate personality traits to driving behavior and accident involvement, finding that honesty-humility, emotionality, agreeableness, conscientiousness, and openness to experience were associated with safer riding, whereas extraversion was associated with riskier patterns, and that safer behavior in turn predicted lower accident involvement [32]. This work is relevant because it locates part of the origin of risky riding in stable individual and psychological dispositions, complementing the error- and motivation-based mechanisms emphasized here and underscoring why risk behavior warrants explicit theoretical structuring rather than treatment as an undifferentiated factor. In Thailand, a survey of 496 riders examined traffic and safety violations directly: factor analysis separated risky behaviors into traffic violations and safety violations, and structural equation modeling confirmed that both were significantly associated with injury severity, with helmetless riding and passenger transport identified as particularly consequential [33]. Taken together, these regional studies show that motorcyclist risk behavior is at once multidimensional and psychologically grounded, and that violations are tied not only to whether a crash occurs but also to the severity of its consequences—evidence that directly motivates modeling risk behavior, protective behavior such as helmet use, and crash involvement within a single framework.

Two themes from this body of work directly shape the current study. The first is the recurring salience of young riders, who combine high exposure, elevated risk behavior, and distinctive responsiveness to interventions; this motivates the generational multigroup analysis. The second is the dual role of safety-equipment use, which appears in the MRBQ both as a behavioral dimension and as a protective factor; this motivates modeling protective behavior as a construct in its own right rather than folding it into the risk factors [8]. The present study extends the tradition by (a) organizing the risk dimensions under explicit theoretical mechanisms, (b) representing them as a second-order construct, and (c) testing risk and protection simultaneously against crash involvement, with formal tests of subgroup invariance.

### 2.3. Human Information Processing and Perceptual and Decision Errors

Human Information Processing (HIP) theory conceives of the rider as a limited-capacity processor who must detect relevant stimuli, perceive and interpret them correctly, decide on a response, and execute it, all under time pressure [34]. Crashes frequently originate at the early, cognitive stages of this sequence: a hazard is not detected in time, a gap is misjudged, or the intentions of another road user are misread. In the present study, these failures are represented by the perceptual and decision errors (PDE) dimension—for example, deviating toward the roadside without noticing pedestrians, failing to anticipate a pedestrian stepping out from behind a parked vehicle, or not anticipating another vehicle cutting in. Such failures have been linked to inattention and distraction during riding and to poorer hazard-prediction ability among less-experienced riders [35,36]. Because these errors reflect lapses in attention and judgment rather than deliberate choice, HIP theory provides the most appropriate lens for understanding them and implies that countermeasures should target hazard perception and situational awareness.

### 2.4. The Generic Error-Modelling System and Vehicle Control Errors

Reason’s Generic Error-Modelling System (GEMS) distinguishes three levels of cognitive control: skill-based behavior, which is automatic and well-practiced; rule-based behavior, which applies learned procedures to familiar situations; and knowledge-based behavior, which is effortful problem-solving in novel situations [37]. Errors can occur at each level—slips and lapses at the skill-based level, and mistakes at the rule- and knowledge-based levels. Vehicle control errors (VCE) in this study—such as braking too late when the vehicle ahead slows, following too closely to stop in an emergency, failing to slow for curves or intersections, or losing control at high speed—map directly onto skill- and rule-based failures in the execution of the riding task, a class of behaviors repeatedly implicated in motorcyclist near-miss and crash events [38,39]. GEMS therefore frames VCE as breakdowns in skilled performance, distinct from the perceptual failures captured by PDE, and suggests that training and skill development are the relevant levers.

### 2.5. The Theory of Planned Behavior, Risky Driving, and Rule Non-Compliance

Whereas errors are unintended, violations are by definition deliberate, and deliberate behavior is best explained by theories of motivated action. The Theory of Planned Behavior (TPB) holds that behavior is driven by intention, which is in turn shaped by attitudes toward the behavior, subjective norms, and perceived behavioral control [40]. Applied to motorcycling, TPB explains why riders choose to engage in risky riding behavior (RDB)—such as running clear red lights, racing from intersections, overtaking in prohibited zones, or riding against traffic—and traffic-rule non-compliance (TRN)—such as failing to stop at zebra crossings, turning onto main roads without checking, or ignoring yield signs. These behaviors are not failures of capacity but expressions of preference under perceived social and situational permission, as shown by studies of why motorcyclists knowingly disregard traffic regulations and of how safety attitudes and risk perception shape the aberrant behavior of commercial riders [41,42]. RDB and TRN are treated as distinct dimensions because the former captures actively dangerous maneuvering while the latter captures the routine disregard of regulatory rules; both, however, share the motivational logic of TPB and respond to interventions targeting attitudes, norms, and enforcement-shaped control beliefs.

### 2.6. A Second-Order Construct of Motorcyclist Risk Behavior

Although PDE, VCE, RDB, and TRN have distinct theoretical origins, they tend to co-occur within individuals: riders who commit more perceptual errors also tend to commit more control errors and more violations [12]. This empirical and conceptual covariation justifies modeling them as four first-order factors that load onto a single second-order construct, motorcyclist risk behavior. The second-order specification captures a general propensity toward unsafe riding while preserving the theoretically meaningful sub-dimensions beneath it. This structure is consistent with the mixed-theory premise: each theory explains one facet, but the facets share a common variance that represents overall behavioral risk. This leads to the study’s first substantive expectation about crash involvement.

**H1.**  *Motorcyclist risk behavior positively affects motorcycle crash involvement*.

### 2.7. Protection Motivation Theory and Protective Behavior

Protective behavior follows a different psychological logic from risk behavior. Protection Motivation Theory (PMT) explains the adoption of self-protective actions as the outcome of two appraisal processes: threat appraisal, in which the individual evaluates the severity of a hazard and their vulnerability to it, and coping appraisal, in which the individual evaluates the efficacy of a protective response and their ability to perform it [43]. When threat and coping appraisals are sufficiently high, protection motivation—and ultimately protective behavior—results. In the motorcycling context, protective behavior (PBE) comprises the use of helmets, gloves, protective jackets or pants, and body armor for elbows, shoulders, and knees; motivational accounts of such safety and stunt-related behavior have begun to be developed and validated for two-wheeler users [44,45]. These behaviors do not prevent crashes from occurring but reduce the probability and severity of injury when they do, and in many jurisdictions they also reflect compliance with safety regulations. PMT predicts that riders who appraise crash threat as severe and protective equipment as effective will adopt such behavior more consistently, and that this behavior should be associated with reduced crash involvement and harm.

The expected negative association between protective behavior and crash involvement warrants fuller theoretical justification, because Protection Motivation Theory positions protective equipment primarily as a means of reducing injury severity once a crash has occurred, rather than as a direct cause of crash avoidance. The relationship posited here is therefore best understood as an indirect, motivationally mediated association rather than a claim of direct physical causation, and it rests on three complementary mechanisms. First, protection motivation is not behavior-specific: the elevated threat and coping appraisals that lead a rider to wear a helmet and protective gear also raise the general salience of crash risk, encouraging more cautious speed choice, greater following distance, and stronger rule compliance, so that protective behavior and lower risk behavior share a common motivational root in a safety-oriented disposition [45]. Second, consistent gear use serves as an observable marker of this latent disposition, such that riders high in protective behavior tend to be systematically lower in the error and violation tendencies that generate crashes [46]. Third, several forms of protective behavior have a plausible conspicuity and harm-limitation pathway to crash occurrence itself: brightly colored helmets and high-visibility clothing increase a rider’s detectability by other road users, and better-protected riders may sustain fewer minor incidents that would otherwise escalate into reportable crashes [38]. These mechanisms are consistent with evidence that helmet and protective-gear use tends to co-occur with safer riding profiles and lower crash and injury rates [47]. Taken together, PMT and the broader safety literature support the expectation that higher protective behavior is associated with lower crash involvement—whether through motivational spillover, dispositional selection, or conspicuity effects—while acknowledging that the pathway is indirect rather than strictly causal. This reasoning yields the second hypothesis.

**H2.**  *Protective behavior is negatively associated with motorcycle crash involvement*.

### 2.8. Heterogeneity Across Rider Subgroups

A model that holds on average may nonetheless operate differently across rider groups, and identifying such differences is essential for targeting interventions. Three grouping variables are theoretically prominent. Gender has been linked to systematic differences in risk-taking and protective behavior, with men generally reporting more violations and women often reporting more cautious profiles, raising the question of whether the behavior–crash relationships differ in strength by gender [41]. Riding experience is associated with the development of skill and hazard perception; more experienced riders may have automated safe responses that change how their behavior translates into crashes, although experience can also breed complacency [48,49]. Generational cohort captures age-linked differences in attitudes, technology use, risk perception, and socialization into traffic culture; younger cohorts in particular have repeatedly been identified as both more prone to risky riding and differently responsive to safety messaging [12,50]. Because theory does not unambiguously predict the direction of these moderations, they are examined as open empirical questions through multigroup analysis rather than as directional hypotheses.

**H3.**  *The structural relationships between behavior and crash involvement differ across rider subgroups defined by (a) gender, (b) riding experience, and (c) generational cohort*.

### 2.9. Self-Reported Crashes as the Outcome Measure

Because the outcome in this study is self-reported crash involvement, the measurement literature on self-reported crashes deserves explicit attention. Self-reports are the most practical way to capture crashes that never enter official records—a large share of motorcycle incidents, especially minor ones, go unreported to police or insurers—and they therefore complement, rather than merely substitute for, archival data [51]. At the same time, self-reports are subject to recall decay, social-desirability bias, and common-method variance, and studies comparing self-report with archival records have found only modest convergence between them [52,53]. Meta-analytic evidence nonetheless shows that traffic offences predict crash involvement, and that self-report measures retain validity as behavioral indicators, particularly among young drivers [54,55], even as reporting biases must be managed [56]. The implication for the present study is twofold: self-reports are appropriate and arguably necessary for capturing the full spectrum of motorcycle crashes, but the design must mitigate bias through clear item wording, a bounded recall window, and validity checks on the measurement model. These considerations inform the methods described next.

## 3. Methods

### 3.1. Research Design

The study employed a cross-sectional, quantitative survey design and analyzed the resulting data with structural equation modeling (SEM), an approach increasingly used to model latent behavioral determinants of crash risk among vulnerable road users [34,57]. A cross-sectional design was appropriate because the aim was to model the associations among latent behavioral constructs and crash involvement in a large, naturally varying population of riders, rather than to manipulate behavior experimentally. SEM was selected because it permits the simultaneous estimation of a measurement model—relating observed questionnaire items to their underlying latent constructs—and a structural model relating those constructs to one another, while accounting for measurement error. The analysis proceeded in three stages: (1) a second-order confirmatory factor analysis (CFA) to validate the structure of motorcyclist risk behavior; (2) a full measurement and structural model to test the hypothesized effects on crash involvement; and (3) multigroup SEM to test the invariance of the structural paths across rider subgroups.

### 3.2. Target Population, Sampling, and Participants

The study targeted regular motorcycle riders in Northeastern Thailand—people who operate a motorcycle routinely within the region. Concentrating on this region reflects the value of locality-specific evidence, since the Northeast differs from other parts of the country in cultural practices, road and traffic infrastructure, and economic conditions, all of which can shape riding behavior and crash exposure. The frame was designed to span riders of differing ages, experience levels, and trip purposes so that the resulting sample would mirror the wider riding community.

Participants were drawn through a stratified, multi-stage procedure that balanced representativeness against field feasibility across four provinces. Districts within each province were first classified as urban or rural; districts were then sampled at random from each stratum in proportion to population size; sub-districts were subsequently selected at random within the chosen districts; and, finally, riders were enrolled through systematic sampling at locations where motorcyclists routinely congregate, such as fuel stations, markets, schools, and workplaces. This design secured geographic spread while keeping data collection manageable over a large study area.

Sample size was set following established guidance for SEM, which calls for enough cases to power parameter estimation in complex measurement and structural models. Applying the widely cited rule of at least ten observations per estimated parameter [58] to the present model implied a target of roughly 4000 respondents, sufficient to detect small-to-medium effects. Fieldwork yielded responses from 4317 riders. Before analysis, the raw responses were screened against pre-specified completeness and eligibility criteria. Cases were excluded if they contained incomplete or missing responses on the study variables (*n* = 620), if the respondent did not meet the eligibility definition of an active motorcycle rider—for example, not currently riding, not having ridden within the recall period, or being under the legal riding age (*n* = 410), if they failed embedded attention or validity check items (*n* = 180), if they exhibited careless or invariant (straight-line) responding (*n* = 120), if they were identified as duplicate submissions (*n* = 50), or if they were flagged as multivariate outliers (*n* = 27). Applying these criteria removed 1407 respondents and retained a final analytic sample of 2910 active riders. Attention or validity checks were embedded items carrying a pre-specified correct response (for example, an instruction to select a designated option); responses that failed these items were treated as invalid. Careless or invariant responding was flagged when a respondent selected the same option across an entire item block (straight-lining) or completed the questionnaire in an implausibly short time relative to the sample median.

This analytic sample of 2910 riders was approximately balanced by gender, with 1431 men (49.2%) and 1479 women (50.8%). Age was distributed across the riding lifespan: 20.6% were 18–24 years old, 37.2% were 25–34, 25.9% were 35–44, 12.3% were 45–54, and 4.0% were 55 or older. In terms of occupation, the sample spanned students (14.7%), government officers (16.8%), private-company employees (18.9%), self-employed and business owners (14.3%), agriculturists (12.5%), general contractors and laborers (15.9%), homemakers (6.5%), and others (0.4%), reflecting a broad cross-section of the riding population rather than a narrow occupational niche. Educational attainment was concentrated below the bachelor’s level (67.0%), with 23.8% holding a bachelor’s degree and 9.2% holding a higher qualification. The large sample size provides ample statistical power for SEM, supports stable estimation of a model with many parameters, and permits meaningful multigroup comparisons. Full demographic details are reported in Table 1.

Crash exposure was substantial in this population. Over the preceding five years, 40.1% of respondents reported never having been involved in a motorcycle crash, while 25.0% reported one crash, 19.7% reported two, 5.9% reported three, 6.9% reported four, and 2.4% reported more than four. In other words, roughly three in five riders had been involved in at least one crash in the past five years, confirming both the high baseline risk of motorcycling in this setting and the suitability of crash involvement as a variable with sufficient variance to model.

### 3.3. Data Collection and Ethical Procedures

Recruitment proceeded through several community channels—fuel stations, markets, educational institutions, government offices, factories, and other public gathering points—to reach riders of varied demographics, trip purposes, and socioeconomic standing. Collection was deliberately spread across different weekdays and times of day so that both weekday commuters and weekend leisure riders were captured, and local leaders and organizations helped reach participants in rural areas where conventional recruitment is less effective. These steps were taken to limit selection bias.

The questionnaire was administered face-to-face by trained research assistants conversant with local dialects and customs. Respondents recorded their own answers to the structured questionnaire, while a research assistant introduced the survey, was available to clarify any items that were unclear, and checked each completed form on submission. This mode was preferred over online or mailed surveys because it remained accessible to respondents with limited literacy or internet access, allowed administrators to clarify item meaning and thereby reduce misinterpretation, and improved data quality by encouraging considered answers and curbing missing responses. Standardized introductions, uniform answers to common queries, and shared procedural guidelines kept administration consistent across assistants and sites.

The protocol was approved by the Institutional Review Board and complied with standards for research involving human participants. All respondents gave informed consent after being briefed on the study’s aims, procedures, risks and benefits, and confidentiality safeguards, and were told that participation was voluntary and could be discontinued at any point without penalty. No personally identifying details were recorded on the instrument, data were stored securely with access limited to the research team, and care was taken to keep the questionnaire and its administration culturally appropriate.

### 3.4. Instrument and Measures

Data were collected with a structured questionnaire, administered face-to-face as described in Section 3.3, that had been reviewed and approved by the relevant research-ethics committee prior to fielding. The questionnaire comprised a demographic section and a behavioral section. The behavioral section operationalized the study’s latent constructs and was adapted from the Motorcycle Rider Behavior Questionnaire (MRBQ) and established protective-behavior items, with wording localized to the study context and refined through expert review [46].

Motorcyclist risk behavior was measured as a second-order construct with four first-order dimensions. Perceptual and decision errors (PDE) were measured with three items capturing failures of hazard detection and anticipation. Vehicle control errors (VCE) were measured with six items capturing failures in braking, following distance, speed regulation, and vehicle handling. Consistent with the MRBQ conceptualization, this dimension also captures functional impairments that degrade real-time control of the machine; the item on riding with impaired vision (e.g., fogged or ill-fitting lenses) is included here because compromised visual monitoring undermines speed regulation, lane-keeping, and gap judgment during riding rather than constituting a discrete hazard-perception failure. Risky riding behavior (RDB) was measured with five items capturing deliberate dangerous maneuvers such as red-light running, racing, illegal overtaking, and wrong-way riding. Traffic-rule non-compliance (TRN) was measured with three items capturing routine disregard of regulatory rules such as failing to yield or to stop for pedestrians. Protective behavior (PBE) was measured with four items capturing the use of protective footwear and clothing, body armor for elbows, shoulders, and knees, gloves, and helmets. Protective behavior is treated as a graded construct spanning actions of differing protective strength: protective footwear and clothing (which chiefly limit abrasion and lower-limb injury), body armor, gloves, and helmets. Footwear and clothing are retained as legitimate, if lower-grade, protective measures—especially relevant in the study’s low- and middle-income riding context—rather than being equated with helmet or armor protection. Motorcycle crash involvement (MCI), the outcome variable, was measured by self-reported involvement in a motorcycle crash over the preceding five years. A five-year recall window was chosen to balance the need to capture enough crash events for stable estimation against the recall decay that affects longer windows [22]. For this item, a crash was defined as any event, while riding or operating a motorcycle, involving a collision or loss of control that resulted in a fall or impact—including collisions with another vehicle, a pedestrian, an animal, or a fixed object, as well as single-vehicle events such as skids or falls. Such events were counted irrespective of injury outcome, property-damage severity, or fault, and respondents reported the number experienced over the preceding five years. Responses were recorded on a six-point ordered scale corresponding to that number (1 = never, 2 = one, 3 = two, 4 = three, 5 = four, 6 = more than four), and, as detailed in Section 3.5, all ordered indicators were treated as continuous in the maximum-likelihood SEM. The full item wording and item-level descriptive statistics are reported in Table 2.

### 3.5. Data Analysis

All structural equation models were estimated using maximum likelihood. Model adequacy was evaluated with a combination of fit indices rather than any single statistic, in line with standard practice [59]. The model chi-square (χ^2^) and its ratio to degrees of freedom (χ^2^/df) were reported, with the recognition that the chi-square statistic is inflated by large samples and will almost always be significant when N is in the thousands; consequently, greater weight was placed on incremental and absolute fit indices that are less sample-sensitive. These included the Comparative Fit Index (CFI) and Tucker–Lewis Index (TLI), for which values at or above approximately 0.95 indicate good fit, together with the Root Mean Square Error of Approximation (RMSEA) and Standardized Root Mean Square Residual (SRMR), for which lower values (RMSEA and SRMR below roughly 0.06–0.08) indicate good fit [58].

Reliability and validity of the measurement model were assessed before interpreting structural relationships. Internal consistency was evaluated with Cronbach’s alpha and composite reliability (CR), with values of 0.70 or above considered acceptable. Convergent validity was evaluated with standardized factor loadings (target ≥ 0.50, preferably ≥0.70) and average variance extracted (AVE ≥ 0.50). Discriminant validity was evaluated using the Fornell–Larcker criterion, which requires the square root of each construct’s AVE to exceed its correlations with all other constructs [60,61]. Only after the measurement model met these criteria were the structural hypotheses (H1 and H2) tested.

Prior to estimation, the data were screened for completeness and for univariate outliers, and the distributional properties of every item were inspected. All indicators were treated as continuous, and the models were estimated with maximum likelihood. This treatment is appropriate here because the ordered response scales contained a sufficient number of categories—six for the crash-involvement outcome (coded 1 = never through 6 = more than four crashes) and five for the behavioral items—a condition under which continuous maximum-likelihood estimation is known to recover parameter estimates and standard errors closely comparable to those obtained from categorical estimators such as diagonally weighted least squares, provided departures from normality are not severe [62,63]. Maximum-likelihood estimation is, moreover, robust to the moderate non-normality typical of behavioral frequency items, particularly at large sample sizes where the sampling distributions of parameter estimates approach normality; the skewness and kurtosis values observed here fell within ranges generally considered acceptable for this estimator. Because the constructs were latent and modeled with multiple indicators, random measurement error in individual items was absorbed into the measurement model rather than biasing the structural estimates, one of the principal advantages of SEM over regression on summed scores.

To confirm that treating the ordered crash-involvement outcome as continuous did not distort the conclusions, two robustness analyses were conducted. Construct scores for motorcyclist risk behavior and protective behavior, derived from the validated measurement model, were regressed on the outcome using (i) an ordered logistic (proportional-odds) model that preserved the six ordered categories of crash involvement, and (ii) a negative binomial model in which the outcome was recoded as the number of crashes over the five-year window (0 = never through 5 = more than four, the highest category being right-censored); the negative binomial specification was preferred over Poisson because the crash count was overdispersed. Both predictors were standardized so that coefficients express change per one standard deviation. To address the possibility that crash involvement reflects riding exposure rather than behavior, both robustness models were additionally estimated with two exposure-relevant covariates recorded in the survey—riding frequency (a three-level measure) and riding experience (years of riding, a proxy for cumulative exposure)—entered alongside the two behavioral construct scores.

Finally, to test H3, multigroup SEM was conducted by splitting the sample by gender (male vs. female), riding experience (1–10 years, *n* = 1181, vs. more than 10 years, *n* = 1729), and generational cohort. Generation was operationalized by age at the time of the survey: Generation Z comprised riders aged 18–29 (corresponding to birth years of approximately 1993–2004; *n* = 1128) and Generation Y riders aged 30–45 (birth years of approximately 1977–1992; *n* = 1366); the oldest cohort (aged 46–61, corresponding to Generation X; *n* = 416) was excluded from the Generation Z–Generation Y contrast. These cohorts were defined by age band at survey rather than by strict canonical birth-year boundaries. Before comparing structural paths, invariance across each grouping variable was examined with a progressive constraint cascade, beginning with a configural (unconstrained) multigroup model and then adding equality constraints in turn on the factor loadings (measurement weights) and on the structural weights, structural covariances, and structural residuals. Invariance at each step was judged by the change in the Comparative Fit Index and the Root Mean Square Error of Approximation, with ΔCFI ≤ 0.01 and ΔRMSEA ≤ 0.015 taken as evidence of invariance, because the χ^2^ difference test is oversensitive at large sample sizes; metric (measurement-weight) invariance, rather than scalar invariance, is the appropriate prerequisite here because the analysis compares structural regression coefficients rather than latent means [64].

## 4. Results

### 4.1. Descriptive Statistics and Reliability

Item-level descriptive statistics are reported in Table 2. Mean ratings on the risk-behavior items were generally low, indicating that most riders reported engaging in errors and violations relatively infrequently: means for the perceptual and decision error items ranged from about 1.48 to 1.56, vehicle control error items from about 1.31 to 1.42, risky riding items from about 1.43 to 1.55, and traffic-rule non-compliance items from about 1.42 to 1.44. Protective-behavior items showed higher and more dispersed means, ranging from about 1.99 to 2.38, reflecting more variable use of helmets and protective equipment across the sample. Skewness values for the behavioral items were positive (most below 2.0) and kurtosis values were modest, indicating departures from perfect normality that are typical of behavioral frequency data but not severe enough to preclude maximum-likelihood estimation in a large sample. The relatively low means on risk items are consistent with a population in which serious violations are the exception rather than the rule, while the more even distribution of protective behavior signals meaningful variation to exploit in the model.

Internal consistency was strong for every construct. Cronbach’s alpha was 0.908 for perceptual and decision errors, 0.929 for vehicle control errors, 0.878 for risky riding behavior, 0.839 for traffic-rule non-compliance, and 0.914 for protective behavior, all comfortably exceeding the 0.70 threshold and consistent with reliabilities reported for MRBQ-type instruments elsewhere [64]. These values indicate that the items within each scale measure their intended construct reliably.

### 4.2. Second-Order Confirmatory Factor Analysis of Motorcyclist Risk Behavior

**Figure 2:**
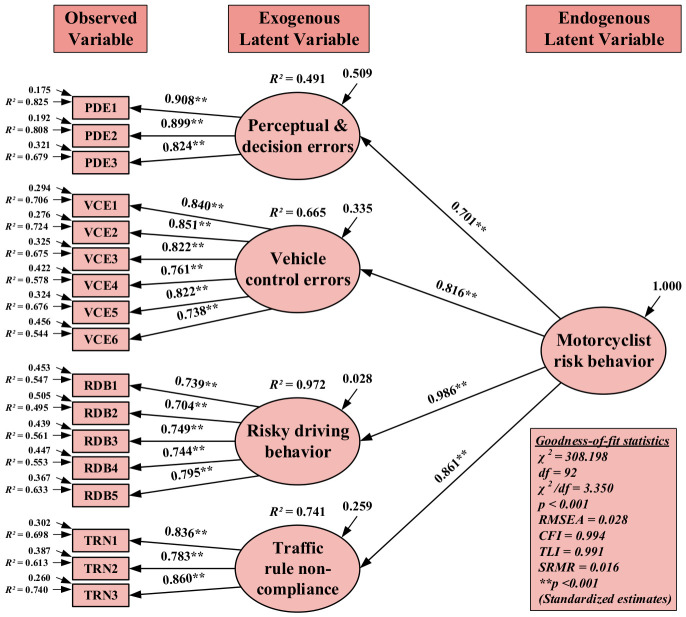
Results of the second-order confirmatory factor analysis of motorcyclist risk behaviors.

The second-order CFA tested whether the four risk dimensions—perceptual and decision errors, vehicle control errors, risky riding behavior, and traffic-rule non-compliance—load onto a single higher-order motorcyclist risk behavior construct (Table 3; Figure 2). The model fit the data well: χ^2^ = 308.198, df = 92, χ^2^/df = 3.350, *p* < 0.001, RMSEA = 0.028, CFI = 0.994, TLI = 0.991, and SRMR = 0.016. The significant chi-square is expected given the large sample and, consistent with standard guidance, was not treated as evidence of poor fit because the RMSEA, SRMR, and incremental indices were uniformly excellent.

At the first-order level, all standardized factor loadings were high and statistically significant (*p* < 0.001), ranging from 0.704 to 0.908, and each construct met the convergent-validity criteria. AVE and composite reliability were 0.771 and 0.910 for perceptual and decision errors, 0.651 and 0.918 for vehicle control errors, 0.558 and 0.863 for risky riding behavior, and 0.684 and 0.866 for traffic-rule non-compliance—every AVE exceeding 0.50 and every CR exceeding 0.70. At the second-order level, all four dimensions loaded strongly and significantly on motorcyclist risk behavior: traffic-rule non-compliance (λ = 0.861), vehicle control errors (λ = 0.816), perceptual and decision errors (λ = 0.701), and, most strongly, risky riding behavior (λ = 0.986), with a second-order AVE of 0.718 and CR of 0.909. The very high loading of risky riding behavior indicates that deliberate dangerous maneuvering is the most central expression of the general risk-behavior propensity, while the lower (though still substantial) loading of perceptual and decision errors indicates that perceptual lapses, though related, are the most distinct of the four facets. These results confirm that the four theoretically derived dimensions cohere into a single, well-defined higher-order construct, supporting the mixed-theory measurement structure.

### 4.3. Measurement Model for the Full SEM

Before testing the structural hypotheses, the full measurement model—adding protective behavior to the second-order risk construct—was estimated (Table 4). All standardized loadings remained high and significant (*p* < 0.001). Convergent validity was satisfactory for every construct, with AVE and CR of 0.753 and 0.901 for perceptual and decision errors, 0.701 and 0.933 for vehicle control errors, 0.650 and 0.903 for risky riding behavior, 0.658 and 0.853 for traffic-rule non-compliance, 0.719 and 0.911 for protective behavior, and 0.652 and 0.881 for the second-order risk construct. The second-order loadings in the full model were again strong (risky riding behavior λ = 0.875, traffic-rule non-compliance λ = 0.837, vehicle control errors λ = 0.805, perceptual and decision errors λ = 0.702), confirming the stability of the higher-order structure when protective behavior is included.

The measurement structure was insensitive to the two items whose assignment might be questioned. Removing the impaired-vision item from vehicle control errors and the footwear-and-clothing item from protective behavior left convergent validity and reliability essentially unchanged (vehicle control errors AVE = 0.71, CR = 0.92; protective behavior AVE = 0.75, CR = 0.90), and re-estimating the structural model without these items reproduced the reported associations (risk behavior IRR = 1.57, protective behavior IRR = 0.84; both *p* < 0.001).

Discriminant validity was supported by the Fornell–Larcker criterion (Table 5). The square root of the AVE on the diagonal exceeded the inter-construct correlations in every case: 0.868 for perceptual and decision errors, 0.837 for vehicle control errors, 0.806 for risky riding behavior, 0.811 for traffic-rule non-compliance, and 0.848 for protective behavior, each larger than its correlations with the other constructs (which ranged from about 0.43 to 0.73). Notably, protective behavior showed only moderate correlations with the risk dimensions (0.429 to 0.535), confirming that protection is empirically distinct from risk rather than its mere mirror image—an important precondition for modeling the two as separate predictors of crash involvement.

The near-perfect second-order loading of risky riding behavior obtained in the risk-only model (λ = 0.986; Section 4.2) does not indicate that this dimension statistically subsumes the higher-order construct. In the full measurement model, the same loading was more moderate (λ = 0.875, R^2^ = 0.766), the remaining three dimensions continued to load strongly (0.702–0.837), and every first-order dimension retained a high, distinct average variance extracted and composite reliability while satisfying the Fornell–Larcker criterion—the square root of the average variance extracted for risky riding behavior (0.806) exceeding its highest inter-construct correlation (0.732, with traffic-rule non-compliance). The four facets are thus empirically separable rather than redundant, and the high loading is best understood as evidence that deliberate risk-taking is the most central, not the sole, expression of the general risk-behavior propensity.

### 4.4. Structural Model and Hypothesis Tests

**Figure 3:**
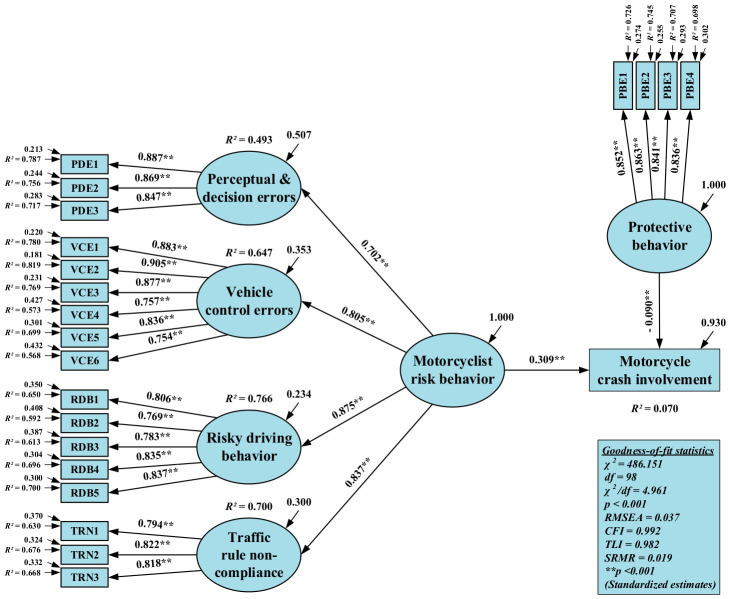
Standardized structural model.

The structural model regressed motorcycle crash involvement on motorcyclist risk behavior and protective behavior simultaneously (Table 6; Figure 3). The model demonstrated excellent fit: χ^2^ = 486.151, df = 98, χ^2^/df = 4.961, *p* < 0.001, RMSEA = 0.037, CFI = 0.992, TLI = 0.982, and SRMR = 0.019; together, the two predictors accounted for R^2^ = 0.070 of the variance in crash involvement. The RMSEA below 0.04 and SRMR below 0.02, with incremental indices near 0.99, indicate that the structural model reproduces the observed covariances very closely.

Both hypotheses were supported. Motorcyclist risk behavior was positively and significantly associated with crash involvement (β = 0.309, SE = 0.025, t = 12.612, *p* < 0.001), supporting H1: riders with a higher general propensity toward errors and violations were substantially more likely to report crash involvement. Protective behavior was negatively and significantly associated with crash involvement (β = −0.090, SE = 0.022, t = −4.173, *p* < 0.001), supporting H2: riders who more consistently used helmets and protective equipment reported lower crash involvement. Because protective equipment principally reduces injury severity rather than preventing crashes, this inverse relationship is interpreted as an indirect, disposition-linked association (Section 2.7) rather than evidence that protective behavior directly lowers crash occurrence. The magnitude of the risk-behavior association was roughly three times that of the protective-behavior association, indicating that, in this population, the harm done by risky riding outweighs the benefit conferred by protective behavior—though both are statistically robust and operate in the expected directions.

The direction and significance of both structural relationships were preserved when crash involvement was modeled as an ordered category or as a count rather than as a continuous variable. In the ordered logistic model, a one-standard-deviation increase in risk behavior was associated with more than double the odds of falling in a higher crash category (OR = 2.41, 95% CI 2.20–2.65, *p* < 0.001), whereas a one-standard-deviation increase in protective behavior was associated with roughly 30% lower odds (OR = 0.70, 95% CI 0.64–0.77, *p* < 0.001). The negative binomial count model showed the same pattern: risk behavior was associated with a higher expected number of crashes (IRR = 1.59, 95% CI 1.52–1.67, *p* < 0.001) and protective behavior with a lower expected number (IRR = 0.83, 95% CI 0.79–0.87, *p* < 0.001). Because these ordered- and count-based specifications reproduce the positive risk association and the negative protective association obtained under maximum likelihood, the continuous treatment of the outcome in the main model does not materially affect the substantive findings (full estimates in Appendix A Table A1).

The behavior–crash relationships were robust to adjustment for the exposure variables available in the data. With riding frequency and riding experience added as covariates, risk behavior remained strongly and positively associated with crash involvement (adjusted OR = 2.41, 95% CI 2.20–2.65; IRR = 1.57, 95% CI 1.50–1.64; both *p* < 0.001) and protective behavior remained significantly negatively associated (adjusted OR = 0.73, 95% CI 0.66–0.80; IRR = 0.85, 95% CI 0.81–0.89; both *p* < 0.001), essentially matching the unadjusted estimates. Riding experience carried an independent positive association (OR = 1.26; IRR = 1.14; *p* < 0.001), consistent with greater cumulative exposure over a longer riding history, whereas riding frequency was not significant (*p* > 0.35); neither covariate attenuated the behavioral associations. The reported behavior–crash relationships therefore do not appear to be an artefact of the exposure differences that could be measured here.

### 4.5. Multigroup Analysis

Multigroup SEM tested whether the two structural paths differed across gender, riding experience, and generational cohort (Table 7).

Before the structural paths were compared, measurement and structural invariance were examined for all three grouping variables using a progressive constraint cascade (Table 8). Across gender, riding experience, and generation, every model in the cascade fit the data well (χ^2^/df between 3.15 and 3.79, CFI ≈ 0.98, TLI ≈ 0.97, RMSEA ≈ 0.027–0.031). Imposing successive equality constraints—first on the factor loadings (measurement weights) and then on the structural weights, structural covariances, and structural residuals—changed fit negligibly at every step, with all ΔCFI ≤ 0.001 and all ΔRMSEA ≤ 0.001, far within the ΔCFI ≤ 0.01 and ΔRMSEA ≤ 0.015 criteria for invariance. Measurement invariance therefore holds, confirming that the constructs are measured on a comparable metric across groups and that the structural-path comparisons reported below are interpretable; the broad structural invariance further indicates that the overall behavioral architecture is largely equivalent across groups. Because this omnibus cascade constrains all structural parameters jointly and is insensitive to a difference confined to a single path, the specific expectations in H3 were tested with focused, single-degree-of-freedom comparisons of the two behavior–crash paths (Table 7), reported next.

For gender, both paths retained their expected signs and significance within each group, but the differences between men and women were not statistically significant. The protective-behavior path was −0.083 (*p* = 0.005) for men and −0.186 (*p* < 0.001) for women, with ΔCMIN = 1.695 (*p* = 0.193); the risk-behavior path was 0.252 for men and 0.310 for women, with ΔCMIN = 3.087 (*p* = 0.079). Although the point estimates suggest that women may derive somewhat more protection from safety behavior and be somewhat more strongly associated with risk behavior, these gender differences did not reach significance, indicating that the behavior–crash relationships are essentially invariant across gender.

For riding experience, the paths were likewise statistically invariant. The protective-behavior path was −0.055 (*p* = 0.084) for riders with 1–10 years of experience and −0.094 (*p* < 0.001) for those with more than 10 years, with ΔCMIN = 1.194 (*p* = 0.275); the risk-behavior path was almost identical across groups (0.277 vs. 0.278), with ΔCMIN = 0.002 (*p* = 0.964). Experience therefore did not change the strength with which risk behavior is associated with crashes, and did not significantly alter the protective association. Among riders with 1–10 years of experience, the protective-behavior coefficient did not reach statistical significance (β = −0.055, *p* = 0.084); this null result should not be interpreted as a confirmed protective effect for that subgroup, nor should the significant effect observed in the full sample and among riders with more than 10 years of experience be generalized to less-experienced riders. Because the moderation test itself was non-significant (ΔCMIN = 1.194, *p* = 0.275), the data provide no reliable evidence that the protective effect genuinely differs by experience; the non-significant subgroup estimate is at least as consistent with limited statistical power in the smaller subgroup as with a true absence of benefit.

For generational cohort, by contrast, both paths differed significantly between Generation Z (*n* = 1128) and Generation Y (*n* = 1366) riders. The protective-behavior path was −0.083 (*p* = 0.010) for Gen Z and −0.193 (*p* < 0.001) for Gen Y, with ΔCMIN = 8.058 (*p* = 0.005); the risk-behavior path was 0.357 (*p* < 0.001) for Gen Z and 0.275 (*p* < 0.001) for Gen Y, with ΔCMIN = 5.239 (*p* = 0.022). These results reveal a clear generational pattern: risk behavior was more strongly associated with crash involvement among younger Gen Z riders, whereas protective behavior was more strongly associated with lower crash involvement among older Gen Y riders. In other words, younger riders are simultaneously more endangered by their risky behavior and less shielded by their protective behavior than their older counterparts. Generation is thus the only one of the three grouping variables that significantly moderates the behavior–crash relationships, providing partial support for H3—specifically H3(c)—while H3(a) for gender and H3(b) for experience were not supported.

## 5. Discussion

### 5.1. The Mixed-Theory Framework and the Structure of Risk Behavior

A central contribution of this study is the demonstration that several behavioral theories can be combined coherently rather than competing. The strong and significant second-order loadings show that perceptual and decision errors (framed by Human Information Processing theory), vehicle control errors (framed by the Generic Error-Modelling System), and the two violation dimensions (framed by the Theory of Planned Behavior) all express a common underlying propensity toward unsafe riding, even though each originates in a different psychological mechanism [23]. The especially high loading of risky riding behavior on the second-order construct suggests that deliberate, motivated risk-taking is the behavioral core of the risk propensity, with errors of perception and control arrayed around it. This does not, however, mean that the construct is reducible to deliberate risky riding. In the full measurement model, the loading was more moderate (λ = 0.875), and perceptual and decision errors, vehicle control errors, and traffic-rule non-compliance all loaded strongly (0.702–0.837) and retained discriminant validity (Section 4.3). The general risk-behavior construct is therefore most strongly expressed by—but not exclusively driven by—deliberate risky riding, indicating that deliberate violations are its most central manifestation rather than its sole content. This pattern reconciles the error and violation traditions: rather than choosing between an error-based account and a motivation-based account of crash behavior, the data indicate that both are facets of a single higher-order construct. For theory, this implies that comprehensive models of motorcyclist behavior should incorporate cognitive, skill-based, and motivational determinants together rather than in isolation.

### 5.2. Risk Behavior and Crash Involvement

The positive association of risk behavior with crash involvement (β = 0.309) is consistent with the substantial international literature linking MRBQ-measured errors and violations to elevated crash risk, and it confirms that the integrated risk construct retains predictive validity [24,25]. Substantively, the result means that riders who more frequently misperceive hazards, mishandle their machines, and deliberately break traffic rules are markedly more likely to crash. Because the construct is anchored in identifiable theoretical mechanisms, the finding also points toward differentiated countermeasures: HIP-based perceptual errors call for hazard-perception training and conspicuity improvements; GEMS-based control errors call for skills training and graduated licensing or on-road coaching [29]; and TPB-based violations call for attitude- and norm-focused campaigns backed by credible enforcement. The dominance of risky riding behavior within the construct suggests that interventions aimed at deliberate violations may yield the largest safety dividends.

### 5.3. Protective Behavior and Crash Involvement

The negative association of protective behavior with crash involvement (β = −0.090) is consistent with the Protection Motivation Theory framing and indicates that protective behavior most plausibly reflects a broader safety-oriented riding profile rather than directly preventing crashes [44]. While helmets and protective gear primarily reduce injury severity, their association with lower crash involvement is consistent with the interpretation that consistent gear use indexes a broader safety-oriented disposition and accompanies more cautious riding. To be explicit about interpretation, the negative association between protective behavior and crash involvement is not read here as evidence that protective equipment directly prevents crashes. It is interpreted instead as primarily reflecting a broader safety-oriented riding profile—a disposition that manifests simultaneously in consistent protective-equipment use and in more cautious riding—with possible secondary contributions from conspicuity and harm-limitation pathways. The protective role of the equipment itself is understood to operate chiefly on injury severity once a crash has occurred, consistent with the Protection Motivation Theory framing in Section 2.7. That protective behavior was empirically distinct from risk behavior—correlating only moderately with the risk dimensions—reinforces the argument that protection and risk are separate behavioral systems, governed by the motivation to protect and the propensity to offend, respectively, and that both must be addressed to improve rider safety. The smaller magnitude of the protective association relative to the risk association indicates that protective behavior, though beneficial, cannot by itself offset the danger posed by risky riding; protection complements but does not substitute for risk reduction.

### 5.4. Generational Moderation and the Limits of Gender and Experience

Perhaps the most actionable finding is the generational pattern. The behavior–crash relationships were invariant across gender and riding experience, suggesting that risk and protection operate similarly for men and women and for newer and seasoned riders—an encouraging sign that the model generalizes across these divisions. Generation, however, mattered. Among Generation Z riders, risk behavior was more strongly associated with crashes, while among Generation Y riders, protective behavior showed a stronger protective association. This double disadvantage for younger riders—more harmed by risk, less helped by protection—aligns with broader evidence that young riders display a high prevalence of risky behavior and elevated crash involvement [14,65], and it suggests that the protective behaviors young riders do adopt may be less consistent or less embedded in a wider safety disposition than those of older riders. The practical implication is that one-size-fits-all interventions are likely to be suboptimal: younger cohorts need interventions that aggressively reduce risky riding, whereas older cohorts may benefit most from reinforcing and sustaining protective habits.

Several developmental and contextual mechanisms may explain why generation, but not raw riding experience, moderates the behavior–crash relationships. Generational cohort captures more than years on the road: it bundles together age-linked maturation of risk appraisal and impulse control, differences in socialization into traffic culture, and contrasting relationships with technology, peer influence, and safety messaging [38]. A younger rider and an older rider may report the same frequency of violations, yet differ in the situations in which those violations occur, the speeds at which they ride, and the secondary precautions they take, so that identical self-reported behavior carries different objective risk. That experience did not moderate the paths—even though more experienced riders showed numerically stronger protective effects—suggests that the safety-relevant differences between younger and older riders are not reducible to skill accumulation alone but reflect broader cohort characteristics. This distinction matters for intervention design, because it implies that simply waiting for young riders to “gain experience” is an insufficient strategy; the cohort-specific vulnerability must be addressed directly through targeted programs.

### 5.5. Relationship to Prior Findings

The results both corroborate and extend the existing motorcyclist-behavior literature. The positive risk–crash association is fully consistent with MRBQ studies across diverse settings that link errors and violations to elevated crash and near-crash involvement, and with naturalistic evidence identifying human error as the leading cause of motorcycle incidents [28]. The prominence of deliberate violations within the risk construct echoes findings that safety violations and speeding are among the strongest behavioral correlates of crashes, particularly among young riders [13]. The generational moderation observed here is consistent with the repeated identification of young riders as a high-risk group and adds a more precise mechanism: it is not merely that young riders behave more dangerously, but that the same level of risk behavior is associated with crashes more strongly for them, while protective behavior buys them less safety. This refinement helps reconcile descriptive reports of high youth crash rates with the behavioral data, and it suggests that age-graded vulnerability operates at the level of the behavior–outcome translation, not only at the level of behavior frequency.

At the same time, the modest convergence between self-report and archival crash data documented in the methodological literature counsels interpretive caution [25,66]. If self-reports systematically under- or over-state crashes, and if such reporting bias varies by age, then part of the observed generational difference could reflect cohort differences in reporting rather than in true crash propensity. Meta-analytic evidence that offences predict crashes more strongly in self-report than in archival data reinforces this caution [54]. The strong measurement properties of the model—high reliability, convergent validity, and clear discriminant validity—mitigate but do not eliminate this concern, and they reinforce the call for multi-source validation in future work.

These associations should also be read against the model’s overall explanatory power. The two behavioral constructs together accounted for about 7% of the variance in crash involvement (R^2^ = 0.070), a modest share that is nonetheless expected, because crash occurrence is multifactorial. Rider behavior is only one input: exposure (distance and time ridden), the road and traffic environment, the behavior of other road users, vehicle and infrastructure characteristics, weather, enforcement intensity, and an irreducible element of chance all contribute to whether a crash occurs, and none of these were modeled here. The framework is therefore best understood as isolating the behavioral contribution to crash involvement rather than as a comprehensive prediction of crash risk, and it captures only part of the crash-risk process. A variance share of this order is, moreover, typical of self-report behavioral models of a relatively rare outcome, and it does not diminish the public-health relevance of the behavioral associations: given the very large exposure base of motorcycling, even a small proportional reduction in behaviorally attributable crashes corresponds to meaningful absolute gains. Fuller accounts of crash risk will require integrating these behavioral constructs with exposure, environmental, and vehicle-level factors in multilevel or multi-source designs.

### 5.6. Theoretical and Practical Implications

Theoretically, the study advances motorcyclist-behavior research by showing that a mixed-theory model integrating HIP, GEMS, TPB, and PMT is not only conceptually coherent but also empirically well-fitting, and that risk and protection can be modeled as distinct but simultaneously operating predictors of crash involvement. This integrative approach offers a template for future behavioral road-safety models that seek to combine the explanatory strengths of multiple theories. Practically, the findings support a two-pronged safety strategy: reducing risk behavior through perception training, skills development, and enforcement-backed attitude change, while promoting protective behavior through threat- and efficacy-focused messaging consistent with PMT. The multigroup results add a targeting dimension, indicating that generationally tailored programs—risk-focused for the young, protection-reinforcing for the older—are likely to be more efficient than uniform campaigns. For policymakers operating with limited budgets, these results provide an evidence base for allocating interventions where they will have the greatest marginal effect [67].

**Figure 4:**
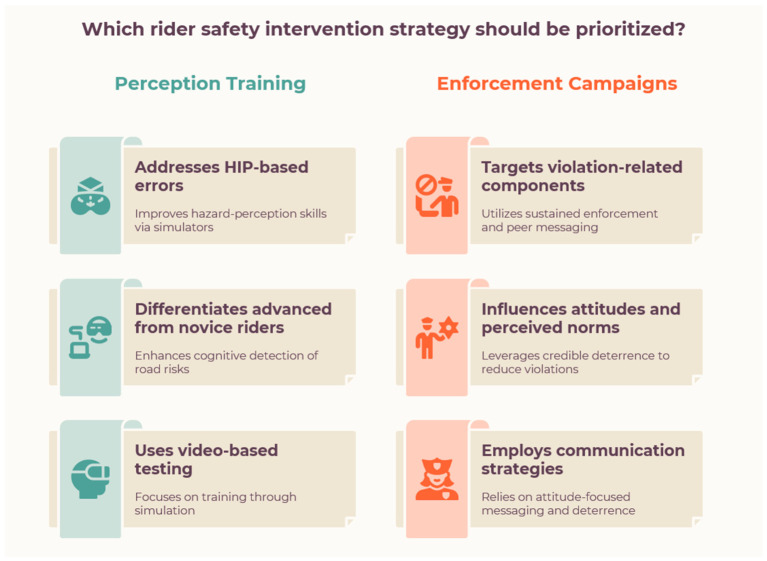
Policy Implications.

Several concrete program designs follow from the model. For the perception-related component, hazard-perception training delivered through simulators or video-based testing—an approach shown elsewhere to differentiate advanced from novice riders—can address the HIP-based errors that the model identifies [29]. For the control-related component, structured skills training and graduated or tiered licensing, which restrict exposure until competence is demonstrated, can address GEMS-based execution failures [48]. For the violation-related component, which carries the heaviest weight in the risk construct, sustained enforcement paired with attitude- and norm-focused communication is indicated, since TPB attributes violations to attitudes, perceived norms, and control beliefs that respond to credible deterrence and peer messaging [41]. For the protective component, PMT-based campaigns that heighten perceived threat severity and vulnerability while reinforcing the efficacy of and self-confidence in using protective equipment can raise consistent gear use. Crucially, the generational findings argue for sequencing and emphasis: programs aimed at younger riders should foreground risk suppression, whereas programs aimed at older riders can lean on reinforcing already-effective protective habits (Figure 4).

### 5.7. Limitations and Future Research

Several limitations qualify these conclusions. First, all constructs—both the behavioral predictors and the crash-involvement outcome—were drawn from the same self-report questionnaire, so the observed associations may be inflated by common-method variance in addition to recall decay and social-desirability bias. Several design features temper this concern: administration was anonymous and interviewer-assisted, reducing social-desirability pressure; the predictors and the outcome used different response formats and referents (behavioral frequency over recent riding versus a five-year count of crash events), which weakens the consistency artefacts that drive common-method bias; and the measurement model displayed clear discriminant validity, with each construct’s average variance extracted exceeding its inter-construct correlations and protective behavior correlating only moderately with the risk dimensions (Table 5)—a pattern inconsistent with a single dominant method factor. These safeguards cannot, however, rule out common-method inflation, and because self-reported crashes converge only modestly with archival records, future work should separate the measurement of predictors and outcomes in time or source and triangulate self-reports with police, hospital, insurance, or telematics data [51]. Second, the crash-involvement measure captured occurrence over a five-year window without distinguishing severity, culpability, or circumstances; richer outcome measures would allow protection and risk to be linked more precisely to crash type and injury. Third, riding exposure was only partially captured. Riding frequency and riding experience were recorded and, when entered as covariates, left the risk and protective associations essentially unchanged (Section 4.4); however, finer exposure metrics—annual mileage, hours spent in traffic, road environment (e.g., urban versus rural), type of motorcycle use, and trip or occupational riding purpose—were not collected, so residual exposure confounding cannot be fully excluded. Because a higher crash count may partly reflect greater distance or time ridden, or riding in more demanding environments, rather than riskier behavior alone, the reported associations are best read as behavior-related rather than fully exposure-adjusted effects, and future studies should record mileage, time in traffic, road environment, and travel purpose and include them as exposure covariates or offsets. Fourth, the study was conducted in a single national context, and although the sample was large and demographically broad, the generalizability of the specific parameter estimates to other riding cultures should be tested through replication. Finally, while the mixed-theory model integrated four theories, it did not directly measure the underlying theoretical antecedents (e.g., attitudes, norms, threat and coping appraisals); incorporating these antecedents would allow a fuller test of the causal pathways the theories propose.

Beyond addressing these limitations, several extensions would enrich the framework. Longitudinal data would permit tests of whether changes in risk and protective behavior over time predict subsequent crash involvement, moving the model from association toward causation. Mediation analyses incorporating the theoretical antecedents—hazard-perception ability for HIP, skill level for GEMS, attitudes and norms for TPB, and threat and coping appraisals for PMT—would clarify the mechanisms linking each construct to behavior, and would allow interventions to be aimed at upstream determinants rather than behaviors alone. Extending the multigroup approach to additional moderators, such as urban versus rural riding environments, occupational riding (e.g., delivery riders) versus personal use, and licensing status, would help identify further high-priority subgroups [42]. Finally, cross-national replication using the same integrated model would test whether the relative weights of the four risk facets and the strength of the protective association are culturally stable or context-dependent, an important question for the international transfer of countermeasures.

## 6. Conclusions

This study examined how motorcyclist risk behavior and protective behavior are jointly associated with motorcycle crash involvement, using a mixed-theory framework that integrates Human Information Processing theory, the Generic Error-Modelling System, the Theory of Planned Behavior, and Protection Motivation Theory within a single structural equation model estimated on a large sample of active riders. The four dimensions of risky riding formed a coherent second-order construct, which was significantly associated with higher crash involvement, while protective behavior was significantly associated with lower crash involvement; the association of risk behavior with crashes was about three times as strong as that of protective behavior. These relationships held across gender and riding experience but varied by generation, with younger riders both more endangered by risk and less shielded by protection than older riders. The findings demonstrate that error-based and motivation-based theories are complementary rather than competing, that protective behavior deserves a place alongside risk behavior in crash-behavior models, and that effective interventions should simultaneously suppress risky riding and reinforce protective habits—while tailoring emphasis to the distinct needs of different generational cohorts. By modeling risk and protection together within an integrated theoretical architecture, the study offers both a more complete account of motorcyclist safety behavior and a practical basis for a two-pronged countermeasure strategy: suppressing risky riding to prevent crashes from occurring, and reinforcing protective behavior to mitigate the injuries that result when they do—the two complementary routes most likely to save lives.

## Figures and Tables

**Table 1:** Demographic data.

Characteristics	Category	Frequency	Percentage
Gender	Male	1431	49.2%
	Female	1479	50.8%
Age	18–24 years old	599	20.6%
	25–34 years old	1083	37.2%
	35–44 years old	753	25.9%
	45–54 years old	357	12.3%
	≥55 years old	118	4.0%
Occupation	Student	428	14.7%
	Government officer	488	16.8%
	Private company employee	551	18.9%
	Self-employed/Business owner	416	14.3%
	Agriculturist	364	12.5%
	General contractor/Laborer	462	15.9%
	Homemaker	188	6.5%
	Others	13	0.4%
Education	Below bachelor’s degree	1950	67.0%
	Bachelor’s degree	692	23.8%
	Above bachelor’s degree	268	9.2%
Motorcycle Crash Involvement In last 5 years	Never	1166	40.1%
	1 time	727	25.0%
	2 times	573	19.7%
	3 times	173	5.9%
	4 times	200	6.9%
	More than 4 times	71	2.4%

Note: N = 2910.

**Table 2:** Statistical summary.

Item	Measures	M	SD	SK	KU
	Perceptual & decision errors (Cronbach’s α = 0.908)				
PDE1	You deviated your vehicle toward the roadside without being cautious of pedestrians walking along the road.	1.480	0.696	1.396	1.545
PDE2	You rode without noticing a pedestrian who might step out from behind a parked vehicle and nearly collided with them.	1.561	0.733	1.223	1.067
PDE3	You rode without noticing that another vehicle might cut in front of you.	1.530	0.722	1.239	0.956
	Vehicle control errors (Cronbach’s α = 0.929)				
VCE1	When the vehicle in front slowed down or braked, you realized too late and had to brake suddenly.	1.418	0.700	1.399	0.546
VCE2	You followed the vehicle ahead too closely, to the extent that in an emergency you would not be able to brake in time.	1.404	0.688	1.440	0.675
VCE3	You did not reduce speed when entering a curve or intersection.	1.399	0.681	1.493	0.999
VCE4	You were unable to control the vehicle when traveling at high speed.	1.309	0.563	1.728	2.336
VCE5	You braked abruptly when encountering a slippery road surface.	1.334	0.608	1.673	1.766
VCE6	You often experienced vision problems (e.g., wearing glasses or foggy lenses) while riding.	1.325	0.633	1.903	2.935
	Risky driving behavior (Cronbach’s α = 0.878)				
RDB1	When the road is clear and empty, you ignore the red traffic signal and proceed immediately.	1.429	0.620	1.168	0.356
RDB2	You race with other vehicles when starting from a signalized intersection.	1.443	0.640	1.175	0.367
RDB3	You overtake in no-overtaking zones (solid-line areas).	1.513	0.679	0.993	−0.141
RDB4	You intentionally ride against traffic or enter a one-way street in the wrong direction.	1.553	0.685	0.884	−0.278
RDB5	You deliberately run a red light.	1.509	0.683	0.999	−0.203
	Traffic rule non-compliance (Cronbach’s α = 0.839)				
TRN1	You do not stop at zebra crossings to allow pedestrians to cross.	1.418	0.623	1.218	0.411
TRN2	You turn left onto a main road without slowing down or checking for oncoming traffic.	1.442	0.641	1.161	0.225
TRN3	You ignore “Yield” signs when entering narrow roads where other vehicles have the right of way.	1.435	0.610	1.109	0.314
	Protective behavior (Cronbach’s α = 0.914)				
PBE1	You wear boots or sneakers, and/or leather pants or protective jackets while riding a motorcycle.	2.109	1.234	0.685	−0.838
PBE2	You wear protective gear for elbows, shoulders, or knees while riding a motorcycle.	1.988	1.185	0.788	−0.784
PBE3	You wear gloves while riding a motorcycle.	2.150	1.310	0.702	−0.897
PBE4	You wear a helmet while riding a motorcycle.	2.381	1.191	0.330	−1.125
	Motorcycle crash involvement (MCI)				
MCI	In the past 5 years, have you been involved in a motorcycle crash?	2.219	1.341	1.038	0.304

**Note:** M = mean, SD = standard deviation, SK = skewness, KU = kurtosis. All behavioral items (PDE, VCE, RDB, TRN, and PBE) were rated on a five-point frequency scale (1 = never, 2 = rarely, 3 = sometimes, 4 = often, 5 = very often). For the risk dimensions (PDE, VCE, RDB, TRN), higher scores indicate more frequent risky behavior; for protective behavior (PBE), higher scores indicate more frequent protective behavior. The motorcycle crash involvement item (MCI) was rated on a six-point scale reflecting the number of crashes over the preceding five years (1 = never, 2 = one, 3 = two, 4 = three, 5 = four, 6 = more than four).

**Table 3:** Confirmatory factor analysis of motorcyclists’ risk behavior.

Constructs and Indicators	Standardized Loading	Standard Error	*t*-Value	*R* ^2^
Perceptual & decision errors	(AVE = 0.771, CR = 0.910)			
PDE1	0.908	0.005	184.210 **	0.825
PDE2	0.899	0.005	176.189 **	0.808
PDE3	0.824	0.007	117.514 **	0.679
Vehicle control errors	(AVE = 0.651, CR = 0.918)			
VCE1	0.840	0.008	103.010 **	0.706
VCE2	0.851	0.008	107.391 **	0.724
VCE3	0.822	0.009	93.430 **	0.675
VCE4	0.761	0.010	76.215 **	0.578
VCE5	0.822	0.008	97.055 **	0.676
VCE6	0.738	0.011	67.990 **	0.544
Risky driving behavior	(AVE = 0.558, CR = 0.863)			
RDB1	0.739	0.010	76.299 **	0.547
RDB2	0.704	0.011	64.839 **	0.495
RDB3	0.749	0.010	77.554 **	0.561
RDB4	0.744	0.010	74.570 **	0.553
RDB5	0.795	0.009	93.138 **	0.633
Traffic rule non-compliance	(AVE = 0.684, CR = 0.866)			
TRN1	0.836	0.010	87.204 **	0.698
TRN2	0.783	0.009	89.858 **	0.613
TRN3	0.860	0.009	100.213 **	0.740
Motorcyclist risk behavior	(AVE = 0.718, CR = 0.909)			
Perceptual & decision errors	0.701	0.012	59.764 **	0.491
Vehicle control errors	0.816	0.009	87.811 **	0.665
Risky driving behavior	0.986	0.007	142.761 **	0.972
Traffic rule non-compliance	0.861	0.009	98.894 **	0.741

Note: ** significant at *p* < 0.001.

**Table 4:** Parameter estimation of the measurement model in SEM.

Constructs and Indicators	Standardized Estimates	S.E.	*t*-Value	*R* ^2^
Perceptual & decision errors (AVE = 0.753, CR = 0.901)				
PDE1	0.887	0.007	133.214 **	0.787
PDE2	0.869	0.007	123.311 **	0.756
PDE3	0.847	0.007	121.484 **	0.717
Vehicle control errors (AVE = 0.701, CR = 0.933)				
VCE1	0.883	0.009	102.747 **	0.780
VCE2	0.905	0.011	84.031 **	0.819
VCE3	0.877	0.012	73.800 **	0.769
VCE4	0.757	0.014	54.113 **	0.573
VCE5	0.836	0.012	72.220 **	0.699
VCE6	0.754	0.010	73.000 **	0.568
Risky driving behavior (AVE = 0.650, CR = 0.903)				
RDB1	0.806	0.010	78.964 **	0.650
RDB2	0.769	0.010	79.699 **	0.592
RDB3	0.783	0.009	85.422 **	0.613
RDB4	0.835	0.009	89.353 **	0.696
RDB5	0.837	0.013	62.661 **	0.700
Traffic rule non-compliance (AVE = 0.658, CR = 0.853)				
TRN1	0.794	0.011	73.999 **	0.630
TRN2	0.822	0.009	89.016 **	0.676
TRN3	0.818	0.010	82.028 **	0.668
Protective behavior (AVE = 0.719, CR = 0.911)				
PBE1	0.852	0.011	78.698 **	0.726
PBE2	0.863	0.011	79.727 **	0.754
PBE3	0.841	0.011	76.955 **	0.707
PBE4	0.836	0.010	84.372 **	0.698
Motorcyclist risk behavior (AVE = 0.652, CR = 0.881)				
Perceptual & decision errors	0.702	0.014	49.507 **	0.493
Vehicle control errors	0.805	0.013	60.150 **	0.647
Risky driving behavior	0.875	0.012	74.429 **	0.766
Traffic rule non-compliance	0.837	0.013	64.975 **	0.700

Note: ** significant at α = 0.001, S.E. denotes Standard error.

**Table 5:** Discriminant validity.

Latent Construct	PDE	VCE	RDB	TRN	PBE
PDE	0.868	0.584	0.614	0.587	0.429
VCE		0.837	0.704	0.673	0.492
RDB			0.806	0.732	0.535
TRN				0.811	0.512
PBE					0.848

Note: Perceptual & decision errors (PDE), Vehicle control errors (VCE), Risky driving behavior (RDB), Traffic rule non-compliance (TRN), and Protective behavior (PBE). The diagonal shows the square root of Average Variance Extracted (AVE).

**Table 6:** The results of the structural model.

Hypothesis Path	Standardized Estimate	Standard Error	*t*-Value	Result
H1: Motorcyclist risk behavior → Motorcycle crash involvement	0.309	0.025	12.612 **	Supported
H2: Protective behavior → Motorcycle crash involvement	−0.090	0.022	−4.173 **	Supported

**Note**: → = regression on, ** significant at *p* < 0.001.

**Table 7:** Multigroup analysis Results.

Factor	Path Difference	Estimate	S.E.	*p*-Value	ΔCMIN (df = 1)	Sig.
Multigroup analysis (Genders)						
	Protective Behavior → Motorcycle crash				1.695	0.1929
Male		−0.083	0.043	0.005		
Female		−0.186	0.040	<0.001		
	Motorcyclist risk behavior → Motorcycle crash				3.087	0.0789
Male		0.252	0.078	<0.001		
Female		0.310	0.080	<0.001		
Multigroup analysis (Riding Experience)						
	Protective Behavior → Motor Crashes				1.194	0.2745
1–10 years		−0.055	0.046	0.084		
>10 years		−0.094	0.040	<0.001		
	Motorcyclist risk behavior → Motorcycle crash				0.002	0.9643
1–10 years		0.277	0.083	<0.001		
>10 years		0.278	0.070	<0.001		
Multigroup analysis (Rider’s Age (Generation))						
	Protective Behavior → Motorcycle crash				8.058	0.0045
Gen Z		−0.083	0.046	0.010		
Gen Y		−0.193	0.040	<0.001		
	Motorcyclist risk behavior → Motorcycle crash				5.239	0.022
Gen Z		0.357	0.087	<0.001		
Gen Y		0.275	0.080	<0.001		

**Table 8:** Measurement invariance tests (multigroup CFA).

Grouping	Model	χ^2^/df	CFI	TLI	RMSEA	RMR	∆CFI	Δ RMSEA
Gender	Unconstrained	3.263	0.982	0.973	0.027	0.026		
	Measurement weights	3.189	0.981	0.974	0.027	0.028	0.001	0.000
	Structural weights	3.173	0.981	0.974	0.027	0.028	0.000	0.000
	Structural covariances	3.173	0.981	0.974	0.027	0.030	0.000	0.000
	Structural residuals	3.147	0.981	0.975	0.026	0.030	0.000	0.001
Riding experience	Unconstrained	3.673	0.978	0.969	0.029	0.031		
	Measurement weights	3.658	0.977	0.969	0.029	0.040	0.001	0.000
	Structural weights	3.652	0.977	0.969	0.029	0.040	0.000	0.000
	Structural covariances	3.785	0.976	0.967	0.030	0.056	0.001	0.001
	Structural residuals	3.748	0.976	0.968	0.030	0.056	0.000	0.000
Gen Z vs. Gen Y	Unconstrained	3.445	0.977	0.967	0.030	0.028		
	Measurement weights	3.390	0.976	0.968	0.030	0.034	0.001	0.000
	Structural weights	3.407	0.976	0.967	0.030	0.035	0.000	0.000
	Structural covariances	3.466	0.975	0.967	0.031	0.059	0.001	0.001
	Structural residuals	3.440	0.975	0.967	0.030	0.059	0.000	0.001

Note: Measurement weights constrain the factor loadings equal across groups; structural weights, structural covariances, and structural residuals impose successive equality constraints on the structural parameters. ΔCFI ≤ 0.01 and ΔRMSEA ≤ 0.015 indicate invariance.

## Data Availability

The data presented in this study are available on request from the corresponding author due to ethical restrictions protecting participant privacy and confidentiality.

## Appendix A

**Table A1:** Robustness analysis of the behavior–crash relationships under ordered and count-based specifications (per 1 SD; N = 2910).

Predictor	Ordered Logistic—OR [95% CI]	Negative Binomial—IRR [95% CI]
Motorcyclist risk behavior	2.41 [2.20, 2.65] ***	1.59 [1.52, 1.67] ***
Protective behavior	0.70 [0.64, 0.77] ***	0.83 [0.79, 0.87] ***

Note: OR = odds ratio; IRR = incidence rate ratio. Both predictors are standardized. Ordered logistic model retains the six ordered crash categories; negative binomial model treats crash count as an overdispersed non-negative count. *** *p* < 0.001.
